# Levels of Urbanization and Parental Education in Relation to the Mortality Risk of Young Children

**DOI:** 10.3390/ijerph120707682

**Published:** 2015-07-08

**Authors:** Hsin-Sheng Fang, Wei-Ling Chen, Chiu-Ying Chen, Chun-Hua Jia, Chung-Yi Li, Wen-Hsuan Hou

**Affiliations:** 1 School of Preclinical Medicine, Beijing University of Chinese Medicine, Beijing 100029, China; E-Mails: oneal0831@yahoo.com.tw (H.-S.F.); jiachunhua125@163.com (C.-H.J.); 2 The Sun Vitality Clinic, Taichung 43748, Taiwan; 3 Department of Health Care Management, National Taipei University of Nursing and Health Sciences, Taipei 10845, Taiwan; E-Mail: ivy2657356@hotmail.com; 4 Department of Public Health, College of Public Health, China Medical University, Taichung 40454, Taiwan; E-Mail: chence@mail.cmu.edu.tw; 5 Department and Institute of Public Health, College of Medicine, National Cheng Kung University, Tainan 70101, Taiwan; 6 Master Program in Long-Term Care, College of Nursing, Taipei Medical University, Taipei 11031, Taiwan; 7 School of Gerontology Health Management, College of Nursing, Taipei Medical University, Taipei 11031, Taiwan; 8 Department of Physical Medicine and Rehabilitation, Taipei Medical University Hospital, Taipei 11031, Taiwan; 9 Center for Evidence-Based Medicine, Taipei Medical University, Taipei 11031, Taiwan

**Keywords:** neonatal mortality, infant mortality, under-five mortality, socioeconomic status, cohort studies

## Abstract

*Background*: The establishment of the National Health Insurance program in Taiwan in 1995 effectively removed the financial barrier to access health care services of Taiwanese people. This population-based cohort study aimed to determine the independent and joint effects of parental education and area urbanization on the mortality risk among children under the universal health insurance coverage in Taiwan since 1995. *Methods*: We linked 1,501,620 births from 1996 to 2000 to the Taiwan Death Registry to estimate the neonatal, infant, and under-five mortality rates, according to the levels of parental education and urbanization of residential areas. We used a logistic regression model that considers data clustering to estimate the independent and joint effects. *Results*: Lower levels of parental education and area urbanization exerted an independent effect of mortality on young children, with a stronger magnitude noted for areas with lower levels of urbanization. Children whose parents had lower levels of education and who were born in areas with lower levels of urbanization experienced the highest risk for neonatal (odds ratio (OR) = 1.60, 95% CI = 1.46–1.76), infant (OR = 1.58, 95% CI = 1.48–1.70), and under-five (OR = 1.71, 95% CI = 1.61–1.82) mortality. *Conclusions*: Even with universal health insurance coverage, lower levels of area urbanization and parental education still exerted independent and joint effects on mortality in young children. This finding implies the inadequate accessibility to health care resources for children from socially disadvantaged families and less urbanized areas.

## 1. Introduction

Parental education, which is a socioeconomic indicator of a family, is strongly associated with children’s morbidity and mortality [1,2,3]. Better nutrition among the children of more educated parents may play a role in curbing such a form of morbidity and mortality [4]. Additionally, children with resourceful parents are healthier at the outset and are more likely to avoid health problems later in life [2]. Parents with more knowledge and higher income are also more capable of utilizing the available resources, for instance, by adhering more closely to recommendations for treating diseases [1,3,5]. However, the extent of inequalities in terms of the socioeconomic position of parents decreased after a child reached the age of 5, and only the 1- to 4-year-old group demonstrated a significant association with parental socioeconomic position [3]. Besides, under-five mortality is a leading indicator of child health and overall development [3].

In addition to family socioeconomic position, the area or neighborhood effects on the mortality or morbidity of an individual have been frequently investigated. An earlier review [6] summarized the potential processes through which a neighborhood can contribute to an individual’s health inequalities. It highlighted the importance of both the physical and social environments of an area. For example, inhabitants living in areas with better food and recreational resources may benefit from healthier lifestyles. A recent research reported that in 22 of the 24 studies reviewed, areas with a lower socioeconomic status (SES) were determined to exhibit higher rates of mortality or prostate cancer incidence [7]. Nevertheless, areas with a higher SES were found to be associated with an increased risk of breast cancer [8,9].

In 1995, the Taiwanese government introduced universal health insurance to cover all residents. The National Health Insurance (NHI) program was intended to ensure the accessibility to health care at a reasonable cost [10]. An observational study revealed that after the introduction of the NHI program, the newly insured used more than twice the amount of outpatient physician visits and hospital admissions than before the implementation of the universal health insurance, bringing those previously uninsured the same amount of health care contacts as the previously insured group. The newly insured also experienced an insignificant increase in emergency department visits [11]. Wen *et al.* conducted a before-and-after comparison of the decade prior to the introduction of the NHI program and the decade after to assess whether its implementation has improved life expectancy and reduced health disparities in Taiwan [12]. They reported that after the introduction of the NHI program, life expectancy increased in health class groups that had higher mortality rates prior to the introduction of the NHI program; at the same time, health disparity narrowed [12]. The NHI program extended the existing insurance coverage from 57% of the population (mostly the employed) to everyone, including children, the elderly, and nonworking adults; consequently, several segments of the population primarily benefited from the program [13].

Lack of insurance has been associated with increased morbidity and mortality [14,15,16], thus, implementation of the NHI program is expected to improve the health of children in Taiwan. In the context of universal health insurance coverage in Taiwan that greatly removes financial barrier to health care, we hypothesized that the risk of under-five mortality in children born after the NHI implementation (*i.e.*, 1996–2000) is associated more with urbanization level than with parental educational attainment, as the later variable may reflect one’s income. Moreover, we also hypothesized that a lower parental educational attainment together with a lesser urbanization may further increase the risk of under-five mortality, as rural area is associated with a lesser accessibility to health care services.

## 2. Methods

### 2.1. Source of Data and Measures of Urbanization and Parental Education

Taiwanese law requires that all live births and deaths be registered within 10 days. Various birth characteristics, including gender, birth weight, gestational age, single/multiple birth, birth order, and place of birth as well as the ages, educational attainment, marital status, and employment of parents, are available for each live birth in the Taiwan Birth Registry (TBR). A total of 1,501,620 live births were identified in TBR between 1996 and 2000. We initially excluded those with missing information on gestational age or with a gestational age of 42 weeks or more. We subsequently employed Tukey’s rule to exclude records with possibly implausible birth weights for gestational age [17] and those with missing birth weight (total *n* = 14,971), consequently yielding 1,486,649 live births for analysis. The TBR has been evaluated and is considered valid and complete [18].

Mortality data were obtained from the Taiwan Death Registry (TDR) for the period from 2000 to 2005. The TDR is regarded as accurate and complete because the registration of all deaths in Taiwan and physicians’ completion of all of the death certificates are mandatory in Taiwan [19].We retrieved the information on date of death and underlying cause of death (UCOD) for each deceased individual.

### 2.2. Study Design, Measurements, and Statistical Analysis

This cohort study design involved 1,486,649 children born in 1996 to 2000. The study cohort was subsequently linked to TDR in 1996–2005, and 11,094 children were found dead at the age of 5 or less. The UCOD for those deceased children was based on the International Classification of Disease Code of the 9th Clinical Modification (ICD-9-CM). The age of death was calculated from the difference between date of death and date of birth.

The primary exposure variables of interest were urbanization level for children’s living areas and parental educational attainment. We categorized each child’s living area at the time of birth into various levels of urbanization based on the TBR information. The basis of this categorization was the classification scheme proposed by Liu *et al.* (2006) who classified all 316 cities and townships of Taiwan into seven ordered levels of urbanization according to various indicators, including population density, proportion of residents with college or higher education, percentage of elderly (>65 years) people, proportion of the agricultural workforce, and number of physicians per 10^5^ people [20]. We further categorized the first three levels of urbanization as higher urbanization, and considered the other four levels as lower urbanization. With regard to the educational attainment of parents, we selected the higher educational level of mother or father to indicate the parent’s educational level for each child. Parent’s educational level of college or higher was regarded as higher educational level, and the educational attainment of elementary or less, junior high, and senior high was considered lower education.

Data analyses initially described the distributions of children according to the four study groups determined by the urbanization level of living area and parent’s educational level, namely, lower urbanization and lower parental education (LULP), lower urbanization and higher parental education (LUHP), higher urbanization and lower parental education (HULP), and higher urbanization and higher parental education (HUHP). We performed statistical test for the interaction of parent’s education with urbanization, and found no such interaction.

Logistic regression models with and without adjustment for potential confounders were consequently used to calculate the crude and adjusted ORs of mortality at <28 days, <1 year, and <5 years after birth. In addition to all of the causes of mortality, we explored the ORs of certain cause-specific mortalities, including infectious and parasitic diseases (ICD-9-CM: 001–139), all neoplasm (140–239), leukemia (204–208), all circulatory diseases (390–459), all respiratory diseases (460–519), congenital anomalies (740–759), conditions originating in the perinatal period (760–779), and injury and poisoning (800–999). The investigation of these diseases in relation to urbanization and parental education was chiefly motivated by their association with SES [1,2,3]. Because the experimental units (*i.e.*, newborn) are naturally clustered in the same family and city/township, deaths of experimental units within a cluster are likely to be correlated. The significance of associations between parent’s education/ urbanization level and children’s mortality was assessed using a multiple logistic regression model with the generalized estimating equation (GEE) method that uses robust standard error estimates to take into account within-family or within city/township correlations of the death event.

Potential confounders considered in the analyses included parental ages, as well as children’s sex, birth order, type of birth, and birth weight standardized for gestational age (*i.e.*, small-for-gestational parental ages (SGA), appropriate-for-gestational age (AGA), and large-for-gestational age (AGA), as the above-mentioned variables were found to be associated with risk of mortality in children [21]. The statistical analyses were performed using the SAS System, version 9.3 (SAS Institute Inc., Cary, NC, USA). Access to both vital statistics and birth registration data was permitted by the Department of Health, Taiwan. Ethical approval from the Institutional Review Board was waived for this analysis because the personal identification numbers are encrypted to protect privacy.

## 3. Results

Table 1 shows the characteristics of the children in this study. On the one hand, the dominance of boys and singletons was observed in the four study groups. Children whose parents had a lower educational level tended to have higher birth orders. On the other hand, the distributions of birth weight, gestational age, and birth weight standardized for gestation were comparable among the four study groups. Parent’s ages were highest in children in the HUHP group, which were some three years older than those of children in the LULP group.

Table 2 shows the ORs of age-specific mortality in relation to lower levels of urbanization or parental education. Compared with children living in higher urbanized areas, those from lower urbanized areas were at significantly increased risk of <28 day mortality with an adjusted OR of 1.34 (95% CI = 1.26–1.42). A lower level of parental education was also associated with a significantly (but with a slightly smaller magnitude) increased risk of <28 day mortality (adjusted OR = 1.28, 95% CI = 1.19–1.37). The logistic regression equation modeling <28 day mortality showed a Nagelkerke R-square of 0.16 and 0.09 for urbanization (area-level variable) and parental education (individual-level variable), respectively Similar findings were observed for <1 year mortality (ORs = 1.32 and 1.28) and <5 year mortality (ORs = 1.39 and 1.34).

Table 3 shows the ORs of mortality at different ages in relation to classifications according to urbanization and parental education. Compared with the children in the HUHP group, the other three groups were all at a significantly elevated adjusted OR of <28 day mortality, with the highest increase noted for children in the LULP group (1.60). The OR was slightly higher for children in the LUHP group than for those in the HULP group (1.31 *vs.* 1.23). Similar findings were observed for both <1 year mortality and <5 year mortality. The adjusted ORs of <1 year mortality were 1.58, 1.25, and 1.22 for children in the LULP, LUHP, and HULP groups, respectively. The corresponding adjusted ORs for <5 year mortality were 1.71, 1.30, and 1.24.

Table 4 shows the ORs of cause-specific <5 year mortality in children in the study groups. Children in the LULP group consistently experienced the highest adjusted OR of various causes of death at the age of five. The most elevated adjusted OR was observed for respiratory diseases (2.97), followed by injury and poisoning (2.41) and leukemia (2.06). The comparison of adjusted ORs between the HUHP and LUHP groups indicated that children in the latter group experienced a significantly higher adjusted OR for most selected causes of death, except infectious/parasitic diseases and circulatory diseases. Moreover, neoplasm (adjusted OR = 1.65), leukemia (adjusted OR = 2.78), and respiratory diseases (adjusted OR = 2.18), were the three causes of death that were associated with increased ORs of <5 year mortality in children in the LUHP group.

**Table 1:** Characteristics of the study subjects.

Variables			Study Groups					
	Lower Urbanization/Lower Parental Education		Lower Urbanization/Higher Parental Education		Higher Urbanization/Lower Parental Education		Higher Urbanization/Higher Parental Education	
	n	%	n	%	n	%	n	%
Gender								
Boy	318,052	52.3	104,564	52.0	205,590	52.1	147,442	52.2
Girl	290,261	47.7	96,375	48.0	189,117	47.9	135,248	47.8
Type of birth								
Singleton	593,877	97.8	195,525	97.4	384,997	97.7	274,102	97.1
Twin	12,440	2.1	4888	2.4	8561	2.2	7844	2.8
≥Triplets	657	0.1	271	0.1	578	0.2	487	0.2
Birth order								
1	248,534	40.9	100,774	50.2	172,196	43.7	142,623	50.5
2	218,292	35.9	74,673	37.2	145,710	36.9	109,162	38.6
3	110,415	18.2	22,477	11.2	62,399	15.8	27,594	9.8
≥4	30,733	5.1	2940	1.5	14,222	3.6	3239	1.2
Gestational wks								
<32	8383	1.4	1973	1.0	4576	1.2	2507	0.9
32–36	37,752	6.2	11,427	5.7	23,622	6.0	16,618	5.9
≥37	562,178	92.4	187,539	93.3	366,509	92.9	263,565	93.2
*x* ± *sd*	38.7 ± 1.8		38.7 ± 1.7		38.7 ± 1.8		38.7 ± 1.7	
Birth weight (g)								
<1500	5918	1.0	1531	0.8	3320	0.8	2032	0.7
1500–2499	35,996	5.9	9833	4.9	22,088	5.6	14,017	5.0
≥2500	566,399	93.1	189,575	94.3	369,299	93.6	266,641	94.3
*x* ± *sd*	3138 ± 478		3167 ± 456		3157 ± 476		3183 ± 463	
SGA ^a^								
Yes	68,208	11.2	17,698	8.8	40,182	10.2	22,335	7.9
No	540,105	88.8	183,241	91.2	354,525	89.8	260,355	92.1
LGA ^b^								
Yes	60,095	9.9	20,750	10.3	41,203	10.4	31,506	11.2
No	548,218	90.1	180,189	89.7	353,504	89.6	251,184	88.9
Maternal age (years)								
<20	44,715	7.5	1729	0.9	18,848	4.8	1587	0.6
20–29	370,177	61.7	103,708	51.8	214,875	54.9	107,349	38.1
30–39	178,910	29.8	92,887	46.4	151,810	38.8	167,481	59.4
≥40	6270	1.0	2086	1.0	6118	1.6	5588	2.0
*x* ± *sd*	26.8 ± 4.9		28.9 ± 4.1		27.9 ± 5.0		30.2 ± 4.1	
Paternal age (years)								
<20	10,842	1.8	178	0.1	4,424	1.1	126	0.0
20–29	234,766	39.0	54,590	27.2	125,681	32.4	47,703	16.9
30–39	320,206	53.2	134,222	66.9	228,879	58.9	206,830	73.4
≥40	36,474	6.1	11,550	5.8	29,473	7.6	27,200	9.7
*x* ± *sd*	30.5 ± 5.6		31.8 ± 4.5		31.4 ± 7.6		33.1 ± 9.7	
Total ^c^	608,313	40.9	200,939	13.5	394,707	26.6	282,690	19.0

Notes: ^a^ SGA: small for gestational age; ^b^ LGA: large for gestational age; ^c^ The inconsistency between total population and population summed for individual variables was attributed to missing information.

**Table 2:** Odds ratio of age-specific mortality according to urbanization or parental education.

Classification	No. of Deaths	(‰)	Unadjusted Estimates		Adjusted Estimates ^a^	
			OR	95% CI	OR	95% CI
<28 day mortality						
Urbanization						
Lower	2890	2.88	1.34	1.26–1.42	1.34	1.26–1.42
Higher	1806	3.73	1.00		1.00	
Parental education						
Lower	3427	3.42	1.30	1.22–1.39	1.28	1.19–1.37
Higher	1269	2.62	1.00		1.00	
<1 year mortality						
Urbanization						
Lower	5241	5.23	1.36	1.30–1.42	1.32	1.26–1.38
Higher	3227	6.67	1.00		1.00	
Parental education						
Lower	6295	6.28	1.40	1.33–1.47	1.28	1.22–1.36
Higher	2173	4.49	1.00		1.00	
<5 year mortality						
Urbanization						
Lower	7045	7.02	1.46	1.41–1.52	1.39	1.34–1.45
Higher	4049	8.37	1.00		1.00	
Parental education						
Lower	8363	8.34	1.48	1.42–1.55	1.34	1.28–1.40
Higher	2731	5.65	1.00		1.00	

Note: ^a^ Adjusted for gender, birth order, type of birth, SGA/LGA, and parental ages.

**Table 3:** Odds ratio of age-specific mortality according to classifications by urbanization and parental education.

Classification	No. of Deaths	(‰)	Unadjusted Estimates			Adjusted Estimates ^a^	
			OR	95% CI		OR	95% CI
<28 day mortality							
Lower urbanization/lower parental education	2289	3.76	1.60	1.46–1.74		1.60	1.46–1.76
Lower urbanization/higher parental education	601	2.99	1.27	1.13–1.41		1.31	1.17–1.46
Higher urbanization/lower parental education	1138	2.88	1.22	1.11–1.34		1.23	1.11–1.35
Higher urbanization/higher parental education	668	2.36	1.00			1.00	
<1 year mortality							
Lower urbanization/lower parental education	4221	6.94	1.71	1.60–1.82		1.58	1.48–1.70
Lower urbanization/higher parental education	1020	5.08	1.25	1.15–1.36		1.25	1.15–1.36
Higher urbanization/lower parental education	2074	5.25	1.29	1.20–1.39		1.22	1.13–1.31
Higher urbanization/higher parental education	1153	4.08	1.00			1.00	
<5 year mortality							
Lower urbanization/lower parental education	5731	9.42	1.89	1.78–2.00		1.71	1.61–1.82
Lower urbanization/higher parental education	1314	6.54	1.31	1.21–1.41		1.30	1.21–1.40
Higher urbanization/lower parental education	2632	6.67	1.33	1.25–1.42		1.24	1.16–1.33
Higher urbanization/higher parental education	1417	5.01	1.00			1.00	

Note: **^a^** Adjusted for gender, birth order, type of birth, SGA/LGA, and parental age.

**Table 4:** Covariate adjusted all causes and cause-specific odds ratio of <5 mortality associated with various disadvantageous groups (Reference group: Higher urbanization/higher parental education).

Causes	Lower Urbanization/Lower Parental Education		Lower Urbanization/Higher Parental Education		Higher Urbanization/Lower Parental Education	
	OR ^a^	95% CI	OR ^a^	95% CI	OR ^a^	95% CI
All causes	1.71	1.61–1.82	1.30	1.21–1.40	1.24	1.16–1.33
Infectious and parasitic diseases (001–139)	1.60	1.12–2.29	0.98	0.61–1.57	1.11	0.75–1.65
Neoplasm (140–239)	1.47	1.00–2.17	1.65	1.06–2.56	0.90	0.58–1.40
Leukemia (204–208)	2.06	1.05–4.04	2.78	1.34–5.75	1.25	0.5–2.64
Circulatory disease (390–459)	1.73	0.62–4.87	1.69	0.51–5.57	1.00	0.31–3.21
Respiratory disease (460–519)	2.97	1.63–5.42	2.18	1.08–4.39	1.56	0.81–3.02
Congenital anomalies (740–759)	1.52	1.36–1.70	1.25	1.09–1.44	1.15	1.02–1.30
Conditions originating in the perinatal period (760–779)	1.79	1.62–1.99	1.40	1.24–1.59	1.34	1.20–1.50
Injury and poisoning (800–999)	2.41	1.99–2.91	1.39	1.09–1.76	1.38	1.12–1.69

Note: **^a^** Adjusted for gender, birth order, type of birth, SGA/LGA, and parental ages.

For children in the HULP group, the significantly increased OR was only observed for congenital anomalies (1.15), conditions originating in the perinatal period (1.34), and injury and poisoning (1.38).

## 4. Discussion

### 4.1. Major Findings

This large-scale cohort study demonstrated the significantly independent and joint effects of parental education and area urbanization on all of the causes of mortality in young children. Given the implementation of the NHI program in Taiwan, our findings suggest that factors other than health care service affordability may be responsible for such individual and area variations in young children’s mortality. The stratified analyses indicated that lower levels of both parental education and area urbanization exerted an independent effect on young children’s mortality, with the effect associated with a lower level of area urbanization demonstrating a greater magnitude.

### 4.2. Strengths and Limitations

This research is the first study in a non-Western context to explore the roles of parental education and area urbanization in determining the mortality risk in young children. The use of a large and representative sample yields reliable and valuable information on individual- and aggregate-level risk factors for young children’s mortality in a setting with a universe health insurance coverage and where no such study has been conducted before. Moreover, we used a statistical method that considers data clustering to isolate the effects of an area on the risk estimates that are associated with the socioeconomic conditions of an individual. 

Despite the aforementioned strengths, our results should be interpreted in light of the limitation that only register-based data were available. We were unable to assess the possible underlying mechanisms through which physical and social environments might have consequences for young inhabitants’ mortality by influencing individual health behavior. Moreover, we used the available variables as proxies for some of the social factors we investigated. For example, a lower level of parental education was employed to indicate poor family SES, and a lower level of urbanization was utilized to signify disadvantaged neighborhoods. Furthermore, we relied on the measurements of individual’s educational attainment and urbanization of residence at one point in time (*i.e.*, time at delivery). We did not examine the cumulative or interacting effects of residential area measured at different times over the life course, the effects related to duration of exposure to certain area environment, and the effects of changes over time in residential environmental neighborhood characteristics. Rauh *et al.* highlighted the need for studies that explore residential history or patterns of exposure to various neighborhood characteristics in examining the area effects on lower birth weight [22].

### 4.3. Interpretations and Implications

Taiwan launched its NHI program in 1995, which largely removed the financial barrier of receiving health care services for the poor [23]. However, the individual and area socio-demographic indicators remained significant predictors for mortality in young children, suggesting possible area and family variations in health care accessibility and utilization of health care services among Taiwanese children. We used mortality data rather than incidence data as the end-point in this study; thus, the concepts of risk and prognosis are mixed [21]. This idea suggests that a higher mortality noted in children with a lower socioeconomic background or from lower urbanized areas could be attributed to a higher incidence of disease, a poor disease prognosis, or both.

Parental SES is inversely associated with mortality risk among children [24,25,26]. However, such an inverse relationship may vary in terms of the gender of children and the cause of mortality. Monden and Smits recently reported that among mothers with a higher level of education, the difference in the mortality chances of boys and girls more closely resembles a “gender neutral” situation than among women with a lower level or lack of education [25]. In other words, girls benefit more than boys from having a more educated mother. Additionally, numerous previous studies established the link between lower parental education and incidence of mortality associated with unintentional injuries [24,27]. Such an observation could be attributed to the fact that injury prevention work against unintentional injuries is less effective among children whose parents have a lower educational level compared with those whose parents have a higher educational level [27]. These findings may explain our result that lower parental education was associated with an increased mortality risk among older (>1 years) children who are vulnerable to unintentional injury. In addition to external causes, the eight-year follow-up study (*i.e.*, from birth to 8 years old) by Kim *et al.* noted that the lower educational attainment of parents was associated with increased mortality risks from respiratory, cardiovascular, and infectious diseases, congenital malformations, and cancer, with the estimated relative risks ranging from 1.43 (cancer) to 2.87 (cardiovascular diseases) [24]. This Korean study also revealed that all of the causes of mortality and in most of the specific causes, the cumulative incidence of mortality rapidly increased until one or two years after birth and then slowed down. However, in external causes and cancer, the cumulative incidence of mortality occurred at a constant pace. The increased risk of mortality from causes other than unintentional injuries in parents with a lower educational level may account for our findings that lower parental education was associated with increased mortality during infancy.

In addition to family SES, neighborhoods were frequently reported to affect individual mortality [6,7]. Our study noted an association between children’s mortality and urbanization level of their residential areas. Certain physical environments may explain our findings. For example, residential segregation by people with higher socioeconomic positions in urban areas may influence the inequality of resources in areas. Better built environment and services and quality of housing may also favor the health of urban residents [7]. Given the NHI program of Taiwan that largely removes the barrier of receiving medical care services, a slight difference exists in the affordability of health care services for people living in various areas. However, accessibility to health care services in the rural parts of Taiwan remains a concern for residents in such areas primarily because of inadequate local medical care resources and the inappropriate utilization pattern of some residents [28]. Nevertheless, examining the role of specific area characteristics is complex because many of the preceding dimensions may be interrelated and may also influence each other.

Mortality variations associated with area conditions are explained at least partly by an individual’s risk factors related to the area. Therefore, previous studies frequently attempted to isolate area influences from individual influences by adopting multilevel analyses that controlled for individual socioeconomic background [29,30]. Pickett and Pearl indicated that studies not adjusting for individual socioeconomic measures were more likely to observe area effects, and that those studies including socioeconomic indicators on the individual level identified a reduced area effect [31]. The study design distinguishes our research from previous studies, as we assessed the independent and joint effects of individual and area conditions on the mortality risk of children. One novelty of this study is that our data revealed that area urbanization provided a relatively larger contribution to children’s mortality than parental education. As previously mentioned, the NHI program launched in 1995 primarily removed the financial barrier to health care services for Taiwanese people. Our findings may imply that family socioeconomic condition has become less imperative in determining the availability of health care services for the children of Taiwan. Therefore, accessibility to rather than affordability of health care services has become increasingly important in determining children’s health. Given the increasing significance of area effect on children’s mortality inequality in the context of Taiwan’s NHI program, our study suggests that investing in less urbanized areas would be beneficial, for example, by providing better housing and infrastructures, as well as improving accessibility to health care services. Such an investment may not only reduce the gap between areas with different levels of urbanization, but also attract higher socioeconomic groups to the less urbanized areas. On the one hand, this approach would increase the positive social influences gained through social interaction; on the other hand, it would increase the attractiveness for more private enterprises, including stores promoting a healthy behavior, to settle in less urbanized areas [7].

Although our study demonstrated a separate and join effects of level of urbanization and parental education on under-five mortality, this observational study was unable to make causal inference on such a link. Specifically, there are several alternatives that might explain the link between urbanization/ parental educational attainment and under-five mortality. For example, a higher mortality from respiratory disease in young children noted in less educated parents could be confounded by certain environmental risk factors (e.g., air pollutants) or perinatal factors (e.g., lower birthweight).

## 5. Conclusions

Lower levels of parental education and area urbanization may exert independent and joint effects on mortality in young children. Children whose parents had lower levels of education and who were born in areas with lower levels of urbanization experienced the highest risk of mortality from birth to 5 years of age, which warrants public health and clinical attention. Even with universal health insurance coverage, lower levels of area urbanization and parental education still exerted independent and joint effects on mortality in young children. This finding implies that accessibility to health care resources for children from socially disadvantaged families and residential areas remains inadequate.
